# Will genetically modified late blight resistant potatoes be the first GM crops to be approved for commercial growing in Norway?

**DOI:** 10.3389/fpls.2023.1137598

**Published:** 2023-03-01

**Authors:** Edward Forbes, Anders Keim Wulff-Vester, Trine (A.K.) Hvoslef-Eide

**Affiliations:** Department of Plant Sciences, Faculty of Biosciences, Norwegian University of Life Sciences (NMBU), Aas, Norway

**Keywords:** *Solanum tuberosum*, *Phytophora infestans* resistance, financial impact, regulatory framework, sustainability, preparedness, CRISPR, Norwegian Gene Technology Act

## Abstract

Last decade’s advances in biotechnology, with the introduction of CRISPR, have challenged the regulatory framework for competent authorities all over the world. Hence, regulatory issues related to gene editing are currently high on the agenda both in the EU and in the European Economic Area (EEA) Agreement country of Norway, particularly with regards to sustainable agriculture. During the negotiations on the EEA Agreement, Norway was allowed to retain three extra aims in the Gene Technology Act: “That the production and use of GMO happens in an ethical way, is beneficial to society and is in accordance with the principle of sustainable development”. We argue the case that taking sustainability into the decisions on regulating gene edited products could be easier in Norway than in the EU because of these extra aims. Late blight is our chosen example, as a devastating disease in potato that is controlled in Norway primarily by high levels of fungicide use. Also, many of these fungicides are being banned due to negative environmental and health effects. The costs of controlling late blight in Norway were calculated in 2006, and since then there have been new cultivars developed, inflation and an outbreak of war in Europe increasing farm input costs. A genetically modified (GM) cisgenic late blight resistant (LBR) potato presents a possible solution that could reduce fungicide use, but this could still be controversial. This paper aims to discuss the advantages and disadvantages of approving the commercial use of a GM LBR potato cultivar in Norway and compare these against currently used late blight management methods and conventional potato resistance breeding. We argue that a possible route for future regulatory framework could build upon the proposal by the Norwegian Biotechnology Advisory Board from 2019, also taking sustainability goals into account. This could favour a positive response from the Competent Authorities without breeching the European Economic Area (EEA) Agreement. Perhaps the EU could adopt a similar approach to fulfil their obligations towards a more sustainable agriculture?

## 1 Background

Today, most nations face food security as being vulnerable, and Norway is potentially the most vulnerable nation concerning food security in Europe. Only 3% of Norwegian land is used for growing food, and of that only 30% is used for grain and 1.4% for potatoes (Flaten and Hisano, 2007; Lombnæs et al., 2011). Over half of the calories consumed in Norway and around 25% of potatoes are imported (Svennerud, 2021; Angelsen and Rebnes, 2022), making Norway highly dependent on the global food supply chain. In the event of a global food crisis, such as drought, war or a severe pandemic, major food exporting countries may significantly reduce export of basic food products, as we saw recently with Ukraine after the Russian invasion (Glauben et al., 2022), and in severe cases Norway could struggle to feed its population.

Potatoes are the fourth most important crop in the world after corn, rice and wheat, and they are an important source of nutrition both globally and in Norway (Lombnæs et al., 2011). The potato plays an important role in sustainably maintaining Norwegian food security, especially under a crisis scenario (Flaten and Hisano, 2007), as potato production is more land and fertiliser efficient per hectare than grain production, potatoes can be produced over the whole of Norway, and tubers are full of important minerals and vitamins and can be stored for long periods of time (Devaux et al., 2014; Store Norske Leksikon, 2021).

The oomycete pathogen *Phytophtora infestans* causes the disease late blight in potato, that threatens potato harvests globally (Hijmans et al., 2000). It is the most significant potato disease in Norway (Sæthre et al., 2006), resulting in high levels of fungicide application with negative effects on human health and the environment. It produces zoospores and sporangia that can travel large distances, as well as overwintering oospores that can survive in soils up to 5 years and act as primary inoculum, making crop rotation as a control less effective (Sæthre et al., 2006). With wetter summers and warmer winters, the effects of climate change in Norway are expected to create more favourable conditions for the spread and infectiousness of *P. infestans* on potato (Cooke et al., 2011).

In 2006, late blight in potato was estimated to cost 55-65 million NOK annually, with fungicides costing farmers on average 22.9 million NOK, application costs 25.6 million NOK, yield losses 5 to 14 million NOK and inspection, research, and advisory services 3.3 million NOK annually (Sæthre et al., 2006). We have recalculated the cost of late blight in Norway to be 105 million NOK before the Ukraine war in 2021, and 125 million NOK after in 2022, considering increased input prices due to inflation and the Ukraine war, as well as the use of modern cultivars, and including VAT of 25% (**Supplementary Table 1**). In addition to this, there can be yield losses from late blight, though these are harder to calculate.

Several popular Norwegian potato varieties, such as ‘Mandel’, are heavily susceptible to late blight, so can only be grown at high altitudes and in Northern Norway where conditions are too harsh for late blight to survive (Roer, 1987; Sæthre et al., 2006). Many of these varieties contain desired traits and have commercial value, so by creating resistant cultivars, they could also be grown in low lying areas with less use of fungicides and with better soil conditions (H A Krogsti, personal conversation, 14 Mar 2022).

Genetic modification (GM) and gene editing (GEd) methods have both been proposed as methods of developing new late blight resistant (LBR) potato cultivars, with an estimated potential to reduce fungicide inputs by over 80% (Kessel et al., 2018). However, the use of GM technology in agriculture is highly controversial in Europe, while China has recently approved another eight new GM crops (ISAAA, 2023).

## 2 Current control methods for *Phytophtora infestans*

Norwegian potato fields were sprayed with fungicide on average 5.6 times a year before 2006 (Sæthre et al., 2006). However, in recent years, this has increased to 8-9 times in a year of heavy infections, even up to 16 times a year, due to more aggressive *P. infestans* strains (B Glorvigen, personal conversation, 15^th^ October 2022). Using fungicides can have severe negative economic, environmental and health effects. Controlling late blight in potatoes can be as much as 25% to 30% of Norway’s entire fungicide usage. Hence, these effects are significant (M Alsheikh, personal conversation, 4th April, 2022). For example, the commonly used fungicide ingredient cymoxanil is suspected to cause birth defects, may cause organ damage over long-term exposure, and is moderately toxic to mammals, honeybees, aquatic organisms and earthworms (Lewis et al., 2016; Plantevernguiden, 2020). Zorvec-Endavia contains the bioactive compound Benthiavalicarb isopentenyl, which has shown carcinogenic potential in two different species (Alvarez et al., 2021). All fungicides used for late blight control have the warning symbol for toxic aquatic effects with long lasting effects (Plantevernguiden, 2020).

Because of these concerns, former common effective fungicides have been banned by the EU and therefore also in Norway (EU regulation, 2020; Saha et al., 2022). As more data is collected on the harmful environmental and health effects of fungicides, pressure is increasing on the EU to continue to ban fungicides, potentially threatening farmer’s ability to chemically control late blight in the EU and Norway (European Regualtion, 2020; Forbond, 2021).

*P. infestans* is notorious for its large genetic variation and ability to constantly mutate and develop resistance to fungicides due to an ability for both asexual and sexual reproduction (Haverkort et al., 2016). Strains with resistance to some fungicide active compounds such as propamocarb, have been found (Lehtinen et al., 2007). In our increasingly globalised world, these mutations spread quickly, making fungicide resistance to *P. infestans* a growing threat to potato production globally.

Plant breeding for LBR is a potential way of reducing need for fungicides against late blight. However, it has many challenges due to the potato’s complex genome, that the potato reproduces primarily by vegetative reproduction making it difficult to cross, and that there is low genetic diversity (Gálvez et al., 2017). Potato is also very susceptible to inbreeding depression (Zhang et al., 2019). Introgression of resistance genes without unwanted effects on the potato genome is difficult due to linkage drag, in addition to the fact that potato is tetraploid, whereas many of its wild relatives containing resistance genes are diploid. Some highly resistant varieties resulting from conventional plant breeding such as ‘Sarpo Mira’ do exist, however these are poorly suited to the Norwegian market and growing conditions (Kim et al., 2012; Gillund et al., 2016; Colon et al., 1995).

## 3 Genetic modification for late blight resistance

Genetic modification presents an alternative to plant breeding without many of the abovementioned issues. Resistance (R) genes from other potato cultivars and wild relatives has to have inserted using traditional GM techniques (Zhu et al., 2012; Witek et al., 2016). In addition, it has been demonstrated that silencing certain susceptibility (S) genes for late blight can increase resistance, however more field trials are necessary to further determine how S gene silencing could affect other crop traits (Sun et al., 2016; Kieu et al., 2021). Multiple R and silenced S genes can be ‘stacked’ in a cultivar to increase the strength and long-term viability of resistance to the pathogen, known as pyramiding (Kim et al., 2012; Sliwka et al., 2012).

One concern regarding GM crops is that introduced genes will escape to wild relatives and have negative ecosystem effects (Quist, 2007). However, *S. tuberosum* is not sexually compatible with either of the two common *Solanum* wild relative species that grow in western Europe: black nightshade (*S.nigrum*) and bittersweet (*S. dulcamara*). This has been demonstrated in studies by Eijlander and Stiekema (1994) and McPartlan and Dale (1994).

Another concern is that cisgenes could spread to other potato growing areas. However, regulations on physical distance and growing intervals between GM and non-GM potato crops and disinfection of machinery can significantly reduce this risk (VKM, 2006; Anastassiadou et al., 2020).

## 4 GM terms and the regulatory framework

The EU defines a GMO as an organism in which “the method of altering genetic material is done in a way that is not natural mating and/or recombination”, and because of this, gene edited organisms using techniques such as CRISPR are regulated as GMO by the EU (Turnbull et al., 2021). However, a proposal that GEd techniques including CRISPR could be regulated separately from GM has been suggested by the Norwegian Biotechnology Advisory Board to the Norwegian government in 2018 (Turnbull et al., 2021). A governemental Committee is currently reviewing the legislation in Norway. As Norway is a member of the European Economic Area (EEA), it follows most EU rules and regulations, and therefore the definitions of GMO as given by EU’s Deliberate Release Directive of 2001 (European Directive 2001; Turnbull et al., 2021). On the other hand, Brexit has lead to a revision of the rules for deliberate release of certain GEd higher plants in England, if the traditional plant breeding techniques (e.g. mutation breeding) could have obtained the same result (UK Practical Law, 2022).

Therefore, following the discovery and use of new breeding techniques (NBTs) including gene editing techniques, there is now a degree of uncertainty regarding what should be legally defined as GMO and if other classifications are necessary (Eckerstorfer et al., 2019). Gene editing is a novel technique involving site directed nucleases (SDN) to make precise incisions or insert DNA sequences at the target DNA area (Turnbull et al., 2021).

In Norway, the use of GM or GEd methods such as CRISPR in agriculture is essentially limited to research use and no GM food crops are grown commercially (Turnbull et al., 2021). However, this may be subject to change as political and consumer pressure to sustainably increase crop yields and adapt to climate change grows, while scientific understanding of gene technologies and their implications advances rapidly (Hjelkrem et al., 2021; Turnbull et al., 2021).

The three additional aims of the Norwegian Gene Technology Act (Norwegian Government, 1993): “That the production and use of GMO happens in an ethical way, is beneficial to society and is in accordance with the principle of sustainable development”, make Norway one of the most restrictive countries in the world for approval of GM crops. However, by demonstrating that the potential GM crop can satisfy all these points, it is more likely that consumers, farmers and industry will support the decision. With the example of the LBR GM potato, this was shown in a workshop by Gillund et al. (2016), and a study by Bioteknologirådet (2020) that showed over 70% of respondents being positive about GM if it would reduce fungicide use and yield losses and thus make agriculture more sustainable.

## 5 Conclusion

In conclusion, there is a strong case for that a LBR GM potato could be the first GM crop to be approved for commercial growing in Norway. Increasing resistance to fungicides, the banning of fungicides by the EU (and Norway), climate change and an increasing focus on Norwegian food self-sufficiency, all create urgent demand for potato cultivars with long lasting and significant LBR. GM and GEd techniques can be used to create potatoes with high levels of LBR in relatively short timeframes that would not otherwise be possible through conventional plant breeding, and therefore present an important potential tool in maintaining food security in Norway in an uncertain future.

It is important that the decision for GM LBR approval in Norway is made based on rational arguments and scientific understanding of its consequences, weighed against the disadvantages of the current control methods and the limitations of potato breeding, and the Norwegian three-part approval system is arguably well adapted to this. Measures can be taken to mitigate the concerns of a GM LBR potato, and arguably the risks of continuing to not use GM in Norway outweighs the risks of allowing it.

## Author contributions

The first author (EF) has done the calculations, under the guidance of the second (AW-V) and the third author (TH-E). All authors contributed to the article and to the writing, and approved the submitted version.
