# Supplementary material for: Will genetically modified late blight resistant potatoes be the first GM crops to be approved for commercial growing in Norway?
Source: Front Plant Sci. 2023 Mar 1;14:1137598. doi: 10.3389/fpls.2023.1137598 (PMC10014530; doi:10.3389/fpls.2023.1137598)
Supplement: Supplementary file 1 [file DataSheet_1.pdf]

Supplementary Table 1. Costs of fungicide applications in potato in Norway, calculated for 2021 (pre-Ukraine war) and 2022, and broken down into the various cost components.

	Approximate tractor driving + average funcigide application + equipment washing costs excluding mva (VAT) pr daa pr year (NOK)	Tractor driving + average funcigide application + equipment washing costs including mva (VAT) pr daa pr year (NOK)	Total area of potato production in Norway excluding Nordland, Troms and Finnmark in 2021 (daa)	Yearly costs of late blight control in Norway estimate (NOK)	Total potato yield in Norway in 2021 (kg)	Average notation price pr kg potet in 2022 (NOK)	Total farmer income from potato production in Norway (NOK)	Late blight control costs as % of income from potato sales at notation price
Before the Ukraine war (2021)	750	938	111,941	104,944,688	368,900,000	6.25	2,305,625,000	4.55
Current (2022)	900	1,125	111,941	125,933,625	368,900,000	6.25	2,305,625,000	5.46
Data source	Bedre Gårdsdrift, Magnus Mo	Bedre Gårdsdrift, Magnus Mo	SSB: https://www.ssb.no/jord-skog-jakt-og-fiskeri/jordbruk/statistikk/potet-og-	Costs pr daa pr year*total area of potato production	SSB: https://www.ssb.no/jord-skog-jakt-og-fiskeri/jordbruk/s	Grøntprodusentene: https://www.grontprodusentene.no/prisinformasjon/Potet/fra/2021/1/til/2021/53	Total potato yield*average notation price	Total farmer income from potato/late blight control costs
